# Molecular classification and outcomes in pediatric aplastic anemia with myeloid neoplasm-associated gene variants

**DOI:** 10.3389/fped.2025.1700402

**Published:** 2025-12-16

**Authors:** Danni Li, Meiling Liao, Yuye Liu, Luying Zhang, Yingxue Hong, Yuxia Guo, Xianmin Guan, Ying Dou, Xianhao Wen

**Affiliations:** Department of Hematology and Oncology, Children’s Hospital of Chongqing Medical University, National Clinical Research Center for Child Health and Disorders, Ministry of Education Key Laboratory of Child Development and Disorders, Chongqing Key Laboratory of Child Rare Diseases in Infection and Immunity, Chongqing, China

**Keywords:** myeloid neoplasm-associated gene variant, aplastic anemia, children, prognosis, molecular classification

## Abstract

**Background:**

The genetic variations in aplastic anemia (AA) patients are closely related to clonal hematopoiesis, but there is limited research on this topic in children with AA. The aim of this study is to investigate the molecular classification and outcomes of children with AA combined with myeloid neoplasm-associated gene variants.

**Methods:**

The clinical features, types of gene variants, mechanisms of action of the mutated genes, and correlations between gene variants and the outcomes of AA patients with myeloid neoplasm-associated gene variants were retrospectively analyzed.

**Results:**

Forty-six AA patients with myeloid neoplasm-associated gene variants were included, and a total of 20 gene variants were identified. The most frequent variant affected *TET2* (9 patients, 19.6%), followed by *ASXL1* (5 patients, 10.9%) and *MPL* (5 patients, 10.9%). Other variants, in descending order, affected *TERT* (4 patients); *SH2B3, FLT3, ETV6,* and *JAK2* (3 patients each); *BCOR, BCORL1, TP53, KIT,* and *SF3B1* (2 patients each); and *CALR, GATA2, RUNX1, CBL, IDH1, IDH2,* and *WT1* (1 patient each). Six patients had 2 gene variants. The original mechanisms of action of the mutated genes mainly involved epigenetics and signal transduction pathways; both groups of genes were affected in 39.1% (18/46) of the patients. The difference in the efficacy of immunosuppressive therapy (IST) among the different gene groups was not significant. Disease severity (*P* = 0.046) and hematological response at 3 months (*P* = 0.002), 6 months (*P* = 0.001), 9 months (*P* = 0.001), and 1 year (*P* = 0.001) were important factors affecting survival time, but genotype was not. None of the patients experienced clonal evolution by the end of the follow-up cut-off time.

**Conclusion:**

In patients with AA combined with myeloid tumor neoplasm-associated gene variants, *TET2, ASXL1* and *MPL* variants were the most frequently observed and primarily involved epigenetics and signal transduction pathways. There was no significant difference in the efficacy of IST among patients with different gene variants. Survival time was associated with disease severity, and the development of a hematological response—particularly when achieved at 3 months—was an independent key factor, whereas genotype was not.

## 1 Introduction

Acquired aplastic anemia (AA) is a disease characterized by immune-mediated bone marrow failure caused by T-cell-mediated destruction of hematopoietic stem and progenitor cells (HSPCs). When immunosuppressive therapy (IST) is used as the first-line treatment for AA patients for whom a suitable donor cannot be identified, the overall survival (OS) rate can reach 80%–85% (1). After hematopoietic function recovery, owing to the relative growth or survival advantages of several somatic variants, AA patients may experience clonal expansion of cells, among whom 15%–20% will experience additional genetic changes and progress to secondary myelodysplastic syndromes (MDS)/acute myeloid leukemia (AML). A prospective study of the late complications of AA revealed that the possibility that AA patients will progress to paroxysmal nocturnal hemoglobinuria (PNH)/MDS/AML increases with time (2).

With the application of next-generation sequencing technology, clonal hematopoiesis can be detected in the hematopoietic stem cells of more than 70% of AA patients, while somatic variants in myeloid neoplasm-associated genes can be detected in approximately 30% of AA patients at the time of diagnosis. These findings suggest that patients with myeloid neoplasm-associated gene variants may be at potential risk of developing MDS/AML, in whom clonal evolution is associated with adverse outcomes (3, 4). However, reports on the characterization of the somatic gene variants that lead to AA clonal hematopoiesis in children with AA are rare (5). This study retrospectively analyzed the clinical features, types, and mechanisms of these gene variants and the correlations between gene variants and outcomes in AA patients with myeloid neoplasm-associated gene variants to provide reference data for the clinical diagnosis and treatment of the disease.

## 2 Patients and methods

### 2.1 Patients

The clinical data of children with AA admitted to our hospital between January 2018 and September 2022 were retrospectively analyzed. The inclusion criteria were as follows: ① the diagnostic criteria of the Diagnosis and Treatment of AA in Children guidelines (2019 edition) (6) alongside the presence of myeloid neoplasm-associated gene mutations; and ② prior IST treatment. The exclusion criteria were as follows: ① no myeloid neoplasm-associated gene mutations; and ② congenital bone marrow failure.

### 2.2 Genetic evaluation

Blood or bone marrow samples were collected from the patients and analyzed with an H030 hematology-oncology panel (designed by Makino), which covers 30 myeloid neoplasm-associated genes—*ASXL1*, *BCOR*, *BCOR1*, *CALR*, *CSF3R*, *EZH2*, *FLT3*, *PIGA*, *GATA2*, *ETV6*, *RUNX1*, *TP53*, *CBL*, *DNMT3A*, *IDH1*, *IDH2*, *JAK2*, *KIT*, *KRAS*, *MPL*, *NRAS*, *SETBP1*, *SF3B1*, *SRSF2*, *SH2B3*, *TET2*, *TERT*, *U2AF1*, *WT1*, and *ZRSR2*. The panel was run by Makino Company. Target gene regions are captured and enriched, followed by sequencing of the captured DNA fragments.The sequencing reads are then aligned to the human reference genome (GRCh37/hg19) for identification of gene mutation sites.

### 2.3 Detection of PNH clones

CD55 and CD59: The absence of CD55 and CD59 on granulocytes and erythrocytes was detected using flow cytometry. The proportions of CD55+ and CD59+ cells were classified as follows: <90%, abnormal; 90%–95%, approximately normal; and >95%, normal.

FLAER: Fluorescein-labeled proaerolysin (FLAER) is an Alexa-488-labeled inactive proaerolysin variant that specifically binds to all the glycated phosphatidylinositides on the granulocyte membrane (GPI) anchor protein. Patients were partially or completely negative for FLAER if the corresponding cell surface anchors were partially or completely absent, respectively, thus preventing or minimizing FLAER binding. If the percentage of FLAER-negative cells was more than 1%, they were considered to be abnormal clones. Testing was completed by Wuhan Kangshengda Medical Laboratory.

### 2.4 Treatment options for IST

All patients were treated orally with cyclosporine A (CsA) at 5 mg/(kg·d) once every 12 h, and the maintenance plasma concentration ranged from 100∼150 ng/mL (trough concentration).

### 2.5 Observation indicators

Efficacy evaluation: In accordance with 2019 guidelines for the Diagnosis and Treatment of AA in Children, efficacy was defined as follows: ① complete remission (CR): ANC > 1.5 × 10^9^/L, Hb > 110 g/L, PLT > 100 × 10^9^/L, independent from red blood cell and platelet transfusion. ② Partial remission (PR): ANC > 0.5 × 10^9^/L, Hb > 80 g/L, PLT >20 × 10^9^/L, independent from red blood cell and platelet transfusion. ③ No remission (NR): inability to achieve the criteria for PR or CR. The patients were followed up at 1, 3, 6, 9, and 12 months after IST, and the cut-off date was December 31, 2023. Early death was defined as death within 6 months after receiving IST, in which case the patient was classified as NR. Pediatric patients who underwent hematopoietic stem cell transplantation within 3–6 months after IST were also classified as NR. Acquisition of a hematological response (HR), including a PR and a CR, was defined as treatment effectiveness.

Clonal evolution was defined as the emergence of new abnormal cytogenetic clones or the transformation of myeloid patterns into MDS or AML.

### 2.6 Statistical methods

Data analysis was performed using SPSS 25.0 software. Measurement data that were not normally distributed are expressed as medians (ranges), and count data are expressed as percentages. Differences were analyzed using the chi-square test and Fisher's exact test. The patients were followed up until December 31, 2023, at which point OS was calculated. The log-rank test was used to assess the difference in survival between the groups. *P* < 0.05 was considered to indicate statistical significance.

## 3 Results

### 3.1 Basic characteristics

From January 2018 to September 2022, a total of 224 patients were diagnosed with AA, among whom 46 patients had myeloid neoplasm-associated gene mutations (46/224, 20.5%). There were 20 males and 26 females in this group; the male to female ratio was 1:1.3. The median age of onset was 5 years and 4 months (ranging from 10 months to 13 years and 11 months), and onset was most common between 3 and 5 years of age. The median duration of the disease was 30 (1–720) days. Twelve patients had nonsevere aplastic anemia (NSAA) (12/46, 26.1%), 16 had severe aplastic anemia (SAA) (16/46, 34.8%), and 18 had very severe aplastic anemia (VSAA) (18/46, 39.1%). Patients mainly sought medical attention due to pallor, fever, bleeding, and abnormal blood counts detected during physical examinations at the onset of the disease. Among them, 4 patients (4/46, 8.7%) had abnormal blood counts detected during physical examinations without typical symptoms. All patients completed bone marrow cytology and bone marrow biopsy examinations. Among them, 40 patients showed varying degrees of hypoplasia, while bone marrow cytology indicated active bone marrow hyperplasia in 6 patients (6/40, 13.0%).Of the 46 patients who underwent bone marrow biopsy, 2 patients showed active hyperplasia with a hematopoietic cell proportion of 40%–50%, and the rest showed hypoplasia. In 6 patients with active hyperplasia indicated by bone marrow cytology, bone marrow biopsy indicated hypoplasia; in 2 patients with active hyperplasia indicated by bone marrow biopsy, cytology indicated hypoplasia. All patients underwent complete chromosomal karyotype analysis, and no abnormal chromosomes were detected.

### 3.2 Genetic evaluation

**Figure 1:**
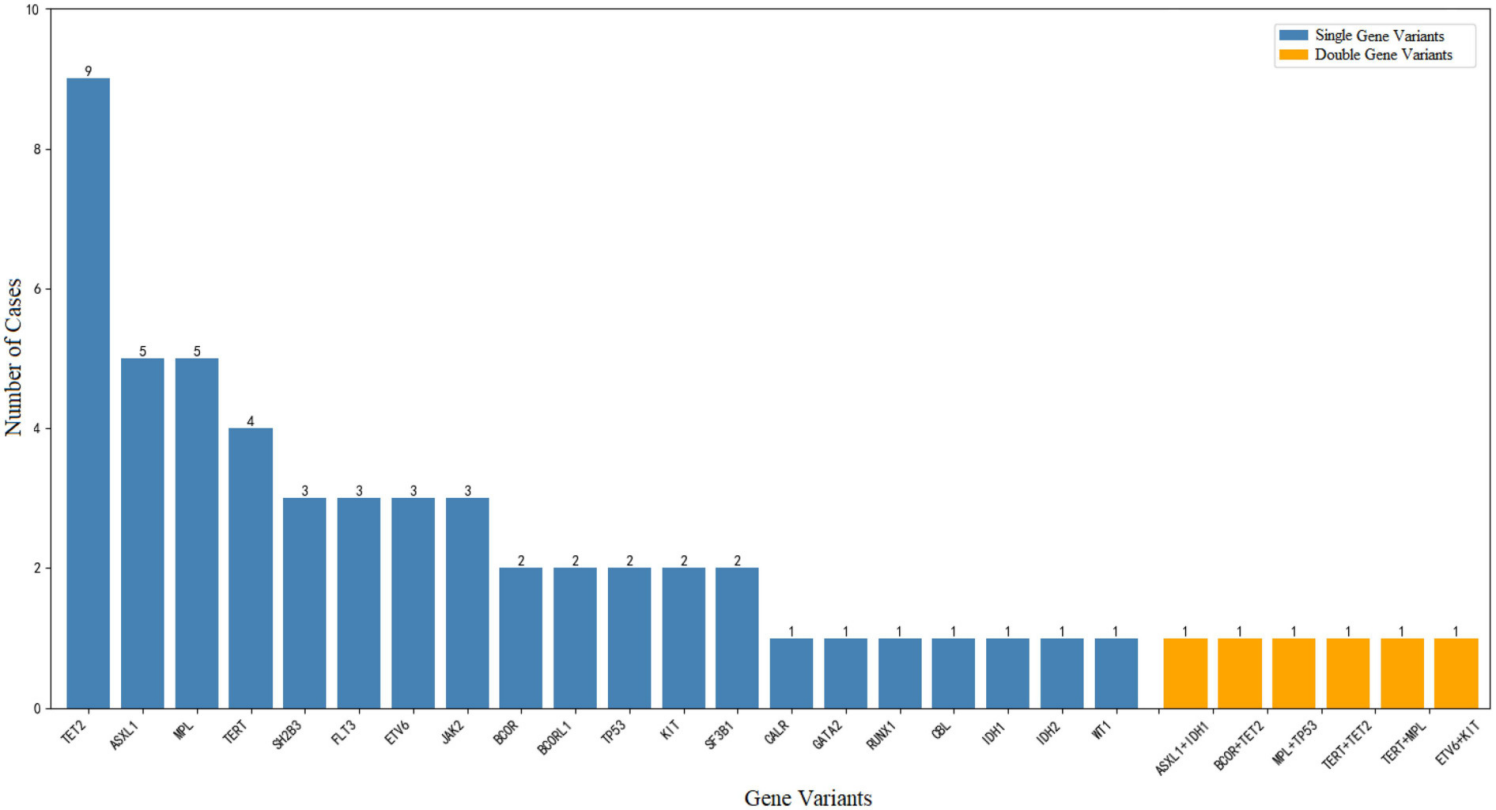
Types of gene variants in myeloid tumors among 46 patients.

Variants in twenty genes, namely, *ASXL1*, *BCOR*, *BCORL1*, *CALR*, *FLT3*, *GATA2*, *ETV6*, *RUNX1*, *TP53*, *CBL*, *IDH1*, *IDH2*, *JAK2*, *KIT*, *MPL*, *SF3B1*, *SH2B3*, *TET2*, *TERT,* and *WT1*, were detected in the 46 patients. The variants detected by gene sequencing examination in 46 patients, including the type of variant, variant allele frequency (VAF), clinical significance, and predicted effect on encoded protein (Table 1). The most frequently mutated gene was *TET2* (9 patients, 19.6%), followed by *ASXL1* (5 patients, 10.9%) and *MPL* (5 patients, 10.9%). In descending order of frequency, the mutated genes included *TERT* (4 patients); *SH2B3*, *FLT3*, *ETV6*, and *JAK2* (3 patients each); and *BCOR*, *BCORL1,TP53*, *KIT*, and *SF3B1* (2 patients each); and *CALR*, *GATA2*, *RUNX1*, *CBL*, *IDH1*, *IDH2*, and *WT1* (1 patient each). Forty patients had only a single gene mutation; the other 6 patients had 2 gene variants: *ASXL1* and *IDH1*, *BCOR* and *TET2*, *MPL* and *TP53*, *TERT* and *TET2*, *TERT* and *MPL,* and *ETV6* and *KIT* (Figure 1).

**Table 1:** Characteristics of gene variants in myeloid tumors among 46 patients.

Patient number	Disease severity	Gene	Gene variant	Chromosomal location	Transcript ID	Exon	Nucleotide variation	Amino acid substitution	Hom/Het	Variant allele frequency (VAF)	Protein function prediction	Pathogenicity analysis	Inheritance pattern	Disease/phenotype
1	SAA	FLT3	STP	chr13:28626700	NM_004119	exon5	c.596G>A	p.C199Y	het	91/72 (0.442)	—	Uncertain	SMu	AML
2	VSAA	IDH2	Epi	chr15:90630671	NM_001289910	exon6	c.659A>G	p.K220R	het	116/118 (0.504)	Benign	Uncertain	AD	D-2-HGA Type2
3	NSAA	JAK2	STP	chr9:5089764	NM_004972	exon20	c.2662A>G	p.T888A	het	238/208 (0.466)	Benign	Uncertain	—	AML
4	SAA	TET2	Epi	chr4:106157703	NM_001127208	exon3	c.2604T>G	p.F868L	hom	2/836 (1)	Benign	Uncertain	—	MDS
5	VSAA	ASXL1	Epi	chr20:31017760	NM_015338	exon7	c.622G>A	p.G208S	het	27/18 (0.4)	Benign	Uncertain	—	MDS
		IDH1	Epi	chr2:209113210	NM_005896	exon4	c.297A>G	p.I99M	het	589/503 (0.46)	Pathogenic	Uncertain	—	GLM1
6	NSAA	SF3B1	SPC	chr2:198268301	NM_012433	exon12	c.1719+8C>T	splicing	het	125/116 (0.48)	—	Uncertain	—	MDS
7	SAA	ASXL1	Epi	chr20:31024131	NM_015338	exon12	c.3616G>A	p.A1206T	het	593/585 (0.5)	Benign	Uncertain	—	MDS
8	SAA	TET2	Epi	chr4:106182959	NM_001127208	exon8	c.3998T>C	p.M1333T	het	192/172 (0.47)	Benign	Uncertain	—	MDS
9	SAA	ASXL1	Epi	chr20:31024839	NM_015338	exon12	c.4324G>A	p.G1442R	het	554/527 (0.49)	Potentially Pathogenic	Uncertain	—	MDS
10	VSAA	JAK2	STP	chr9:5022088	NM_004972	exon3	c.101A>G	p.Q34R	het	415/384 (0.48)	Benign	Uncertain	—	AML
11	SAA	BCOR	Epi	chrX:12923756	NM_001123385	exon4	c.1024C>T	p.R342X	het	—	—	Uncertain	—	CMML
		TET2	Epi	chr4:106157539	NM_001127208	exon3	c.2440C>T	p.R814C	het	—	Likely Benign	Uncertain	—	MDS
12	VSAA	MPL	STP	chr1:43806148	NM_005373	exon6	c.944T>G	p.F315C	het	114/82 (0.42)	Pathogenic	Uncertain	—	MF
		TP53	TRF	chr17:7579705	NM_000546	exon3	c.91G>A	p.V31I	het	158/163 (0.51)	Potentially Pathogenic	Uncertain	AD	BMFS5
13	NSAA	ASXL1	Epi	chr20:31024423	NM_015338	exon12	c.3908A>G	p.K1303R	het	131/133 (0.5)	Benign	Uncertain	—	MDS
14	SAA	TET2	Epi	chr4:106157703	NM_001127208	exon3	c.2604T>G	p.F868L	het	—	—	uncertain	—	MDS
15	VSAA	ETV6	TRF	chr12:12006411	NM_001987	exon4	c.379C>T	p.R127W	het	158/142 (0.47)	—	Uncertain	—	AML
16	SAA	MPL	STP	chr1:43806154	NM_005373	exon4	c.944T>G	p.F315C	het	—	Pathogenic	Uncertain	—	MF
17	VSAA	TERT	TRF	chr5:1258665	NM_198253	exon13	c.3032+48C>T	splicing	het	52/50 (0.49)	—	Uncertain	AD	TRBMF
		TET2	Epi	chr4:106158550	NM_017628	exon3	c.3451G>T	p.E1151X	het	493/474 (0.49)	—	Uncertain	—	MDS
18	NSAA	SF3B1	SPC	chr22:30733853	NM_005877	exon12	c.1777G>A	p.A593T	het	433/458 (0.514)	Benign	Uncertain	—	MDS
19	NSAA	MPL	STP	chr1:43804962-43804963	NM_005373	exon4	c.413delT	p.I138Tfs*28	het	26/27 (0.51)	—	Pathogenic	—	MF
20	VSAA	TET2	Epi	chr4:106157539	NM_001127208	exon3	c.2440C>T	p.R814C	het	323/341 (0.51)	Likely Benign	Uncertain	—	MDS
21	VSAA	FLT3	STP	chr13:28622518	NM_004119	exon9	c.1099T>A	p.F367I	het	150/114 (0.43)	—	Uncertain	—	AML
22	VSAA	TP53	TRF	chr17:7579705	NM_000546	exon3	c.91G>A	p.V31I	het	—	Potentially Pathogenic	Uncertain	AD	BMFS5
23	VSAA	TET2	Epi	chr4:106157539	NM_001127208	exon3	c.2440C>T	p.R814C	het	342/372 (0.52)	—	Pathogenic	—	MF
24	NSAA	SH2B3	STP	chr12:111884942	NM_005475	exon5	c.940G>A	p.E314K	het	42/50 (0.543)	Benign	Uncertain	SMu	MF
25	VSAA	SH2B3	STP	chr12:111884942	NM_005475	exon5	c.940G>A	p.E314K	het	253/280 (0.53)	Benign	Uncertain	SMu	MF
26	NSAA	JAK2	STP	chr9:5090505	NM_004972	exon21	c.2821C>G	p.L941V	het	58/56 (0.491)	Pathogenic	Uncertain	SMu	AML
27	NSAA	TERT	TRF	chr5:1293449	NM_198253	exon2	c.1552G>A	p.A518T	het	103/92 (0.472)	—	Uncertain	AD	TRBMF
28	SAA	BCORL1	Epi	chrX:129147217	NM_021946	exon3	c.469G>A	p.A157T	het	728/699 (0.49)	Benign	Uncertain	—	MDS
29	SAA	GATA2	TRF	chr3:128205688	NM_032638	exon2	c.187C>G	p.P63A	het	153/154 (0.502)	Benign	Uncertain	—	MDS
30	VSAA	RUNX1	TRF	chr21:36164771-36164771	NM_001754	exon9	c.1098_1103dupCGGCAT	p.I366_G367dupIG	het	64/56 (0.47)	—	Uncertain	SMu, AD	AML
31	NSAA	KIT	STP	chr4:55598063	NM_000222	exon16	c.2260C>T	p.P754S	het	32/51 (0.61)	Benign	Uncertain	—	AML
32	VSAA	TET2	Epi	chr4:106158550	NM_017628	exon3	c.3451G>T	p.E1151X	het	276/232 (0.46)	—	Uncertain	—	MDS
33	VSAA	FLT3	STP	chr13:28626698	NM_004119	exon5	c.598G>A	p.D200N	het	234/240 (0.51)	Benign	Uncertain	—	AML
34	SAA	CALR	STP	chr19:13054347	NM_004343	exon8	c.961-4C>A	splicing	het	404/358 (0.47)	—	Uncertain	SMu	MF
35	NSAA	ASXL1	Epi	chr20:31022410	NM_015338	exon12	c.1895G>A	p.C632Y	het	248/266 (0.52)	Benign	Uncertain	—	MDS
36	SAA	MPL	STP	chr1:43805736-43805737	NM_005373	exon5	c.793delC	p.L265fs*12p.C93S	het	146/125 (0.46)	—	Likely pathogenic	—	MF
		MPL	STP	chr1:43804278	NM_005373	exon3	c.278G>C	p.C93S	het	347/377 (0.52)	Pathogenic	Uncertain	—	MF
37	NSAA	BCORL1	Epi	chrX:129147859	NM_021946	exon4	c.1111A>G	p.T371A	het	11/9 (0.45)	Benign	Uncertain	—	MDS
38	VSAA	WT1	TRF	chr11:32456490-32456490	NM_024426	exon1	c.405_416dupACCCCCGCCGCC	p.P138_P141dupPPPP	het	31/19 (0.38)	—	Uncertain	SMu, AD	WT
39	NSAA	TERT	TRF	chr5:1294664-1294665	NM_198253.3	exon2	c.336del	p.Glu113ArgfsTer15	het	50/42 (0.46)	—	Pathogenic	—	TRBMF
		MPL	STP	chr1:43815051	NM_005373.3	intron10	c.1565+21G>A	—	het	74/98 (0.57)	Benign	Uncertain	—	MF
40	VSAA	SH2B3	STP	chr12:111856623	NM_005475.3	exon2	c.674G>C	p.Arg225Pro	het	25/23 (0.48)	Uncertain	Uncertain	SMu	MF
41	SAA	TET2	Epi	chr4:106156867	NM_001127208	exon3	c.1768C>A	p.L590I	het	64/62 (0.49)	Likely Benign	Uncertain	—	MDS
42	SAA	TERT	TRF	chr5:1258665	NM_198253	exon1	c.137C>G	p.A16G	het	—	—	Uncertain	AD	TRBMF
43	SAA	ETV6	TRF	chr12:12006411	NM_001987	exon5	c.818C>T	p.T273M	het	—	—	Uncertain	—	AML
44	VSAA	BCOR	Epi	chrX:12923756	NM_001123385	exon4	c.2582G>A	p.R861H	het	—	—	Uncertain	—	CMML
45	SAA	ETV6	TRF	chr12:12006402	NM_001987	exon4	c.370A>G	p.R124G	het	204/254 (0.55)	Potentially Pathogenic	Uncertain	—	AML
		KIT	STP	chr4:55592103	NM_000222	exon9	c.1427G>C	p.S476T	het	251/282 (0.53)	Benign	Uncertain	—	AML
46	VSAA	CBL	STP	chr11:119149244	NM_005188	exon9	c.1252T>C	p.F418L	het	110/111(0.5)	Pathogenic	Pathogenic	AD,SMu	JMML

Het, heterozygous; Hom, homozygous; SMu, somatic mutation; AD, autosomal dominant; AML, acute myeloid leukemia; D-2-HGA Type2, D-2-Hydroxyglutaric Aciduria Type 2; MDS, myelodysplastic syndromes; GLM1, glioma susceptibility 1; CMML, chronic myelomonocytic leukemia; MF, myelofibrosis with somatic mutation; BMFS5, bone marrow failure syndrome type 5; TRBMF, telomere-associated myeloid hematopoietic failure; WT, wilms tumor; JMML, juvenile myelomonocytic leukemia.

**Figure 2:**
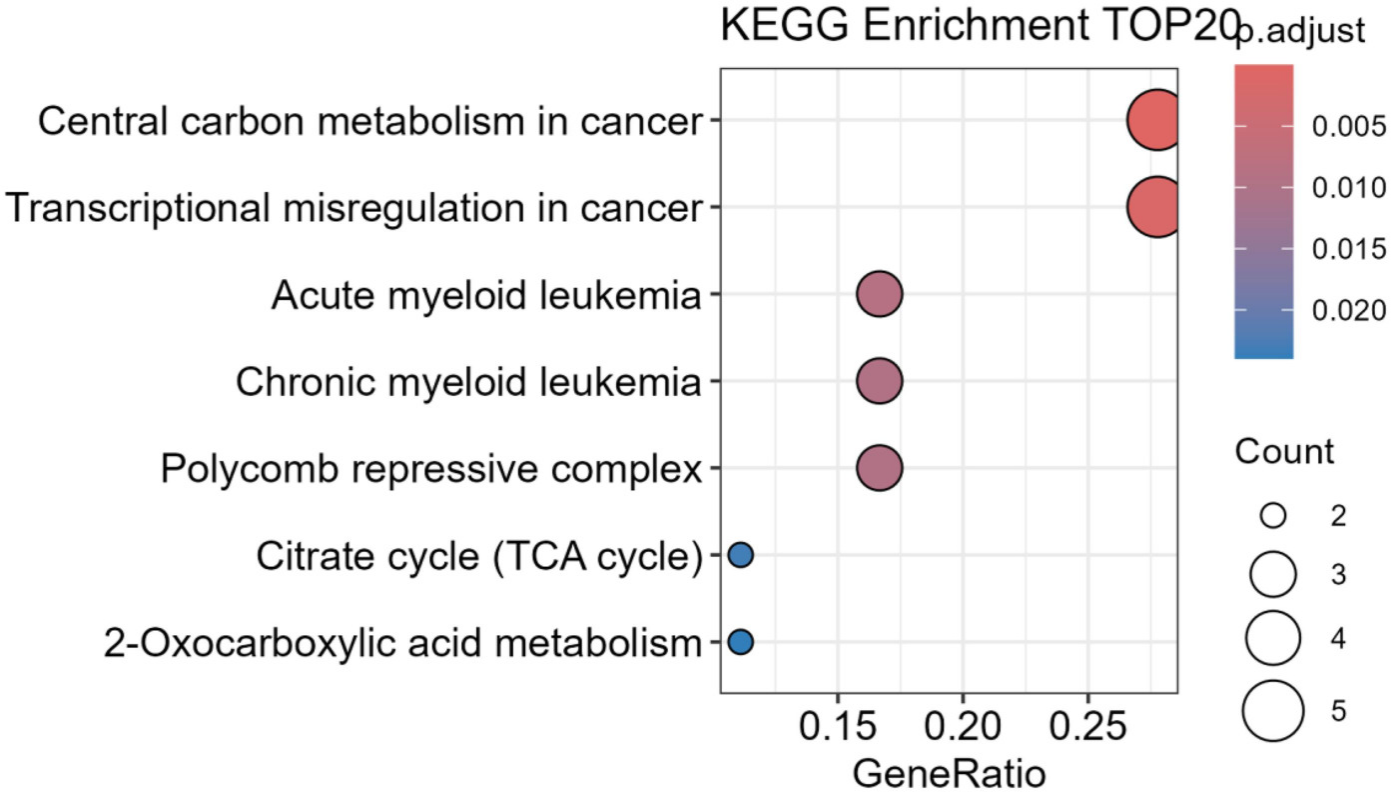
KEGG enrichment analysis of 20 genes.

Based on the genes' mechanisms of action, the 20 mutated genes were divided into epigenetic genes, transcription regulator genes, signal transduction pathway genes, and spliceosome-related genes. The epigenetic genes included ASXL1, TET2, BCOR, BCORL1, IDH1, and IDH2. The transcription regulator genes were RUNX1, ETV6, TERT, TP53, GATA2, and WT1. The signal transduction pathway genes included JAK2, MPL, SH2B3, FLT3, CALR, CBL, and KIT. SF3B1 is the sole spliceosome-related gene. The genes associated with epigenetics and signal transduction pathways were the main genes. The KEGG database was used to perform pathway enrichment analysis on 20 genes, and the results showed that these genes are mainly involved in tumor related metabolic and transcriptional regulatory pathways. The main pathways involved are central carbon metabolism in cancer, transcriptional misregulation in cancer, AML, chronic myeloid leukemia, polycomb repressive complex, citrate cycle (TCA cycle), and 2-Oxocarboxylic acid metabolism (Figure 2).

There was no statistical significance between gene mutations and baseline blood parameters (white blood cells, *P* = 0.086; absolute neutrophil count, *P* = 0.064; absolute lymphocyte count, *P* = 0.562; red blood cells, *P* = 0.276; hemoglobin, *P* = 0.091; platelets, *P* = 0.406; reticulocyte count, *P* = 0.189).There was no significant difference between the different types of gene variants and the sex, age, disease course, or severity of the disease (Table 2). The reduction in sample size in the figure is due to patients undergoing hematopoietic stem cell transplantation (HSCT), death, and loss to follow-up. These cases were directly excluded to conduct hematological assessments at each time point, aiming to determine whether there were statistical differences in response rates between the groups.

**Table 2:** Clinical characteristics among different types of gene variants.

Variables		Epi	TRF	STP	SPC	Two types	*χ* ^2^	*P*
Gender	Male (*n* = 20)	5	4	8	1	2	2.635	0.682
	Female (*n* = 26)	12	4	7	1	2		
Age	0–3 y (*n* = 3)	1	0	2	0	0	8.060	0.374
	3–5 y (*n* = 30)	14	5	7	2	2		
	>5 y (*n* = 13)	2	3	6	0	2		
Course of disease	<1 mo. (*n* = 20)	6	4	7	1	2	4.320	0.889
	1 mo.–3 mo. (*n* = 19)	9	3	4	1	2		
	≥3 mo. (*n* = 7)	2	1	4	0	0		
Degree of BMF	NSAA (*n* = 12)	3	1	5	2	1	6.695	0.584
	SAA (*n* = 16)	8	3	4	0	1		
	VSAA (*n* = 18)	6	4	6	0	2		

Epi, epigenetics; TRF, transcriptional regulatory factor; STP, signal transduction pathway; SPC, spliceosome; two types, Two different types of gene mutations.

### 3.3 PNH detection

Forty-one patients underwent PNH clonal detection. In 4 patients, the CD55/CD59 ratio was between 90% and 95%, indicating approximately normal levels. The levels were normal in the remaining 37 patients. The percentage of FLAER-negative cells was >1% for two patients; thus, they were considered to harbor abnormal clones. One patient exhibited a monocyte clone percentage of 4.65% and a granulocyte clone percentage of 5.77%; this patient carried an *FLT3* variant. Another patient exhibited a monocyte clone percentage of 0.67% and a granulocyte clone percentage of 0.91%; this patient carried an *SH2B3* variant.

### 3.4 Evaluation of efficacy

Event-Free Survival (EFS) is defined as the time from diagnosis to the first event at the last follow-up. An event is defined as PR or CR. The overall effective rates for 1, 3, 6, 9, and 12 months of IST treatment were 7.1% (3/42), 33.3% (12/36), 48.1% (13/27), 66.7% (14/42), and 71.4% (15/21), respectively. There was no significant difference in the total effective rate of different degrees of AA at the time points noted above (Supplementary Table 1). Sixteen patients received allogeneic hematopoietic stem cell transplantation (HSCT). Of these patients, 11 underwent HSCT in our hospital, and 5 underwent HSCT in other hospitals. The median time from diagnosis to HSCT was 6 months (3.0–7.8 months). Two patients died after transplantation: 1 patient had severe intestinal GVHD, 1 patient had refractory septic shock and heart failure. The remaining patients survived and achieved CR.

Among the 46 patients, 40 had single gene variants (40/46, 86.9%). For 9 patients with *TET2* variants, the effective rates at 3 and 6 months were 37.5% (3/8) and 42.9% (3/7), respectively. Of the 5 patients with *ASXL1* variants, 1 also had an *IDH1* variant. This patient died of septic shock 9 months after diagnosis. of the 5 patients with *MPL* variants, 2 patients had 2 gene variants. Significant differences in the platelet count (*P* = 0.053) or megakaryocyte count in the bone marrow (*P* = 0.567) were not observed between patients with *MPL* variants and those with other gene variants. There was no significant difference in the hematological reaction rate after IST in patients with different genotypes (Table 3). Differences in sample size occurred because patients underwent HSCT, died, or were lost to follow-up during treatment. Among the 40 patients with single-gene variants, 12 underwent HSCT, including 5 patients with single-gene variants in epigenetic regulators (3 with TET2 variants, 1 with IDH2 variant, 1 with ASXL1 variant), 2 with variants in transcriptional regulators (TP53, ETV6), and 5 with variants in signal transduction genes (SH2B3, CALR, CBL, MPL, KIT).

**Table 3:** Hematologic response among different types of gene variants.

Variable	Epi	TRF	STP	SPC	χ²	*P*
1mo.	1/14 (7.1%)	0/6 (0.0%)	1/14 (7.1%)	1/2 (50.0%)	3.827	0.825
3mo.	6/12 (50.0%)	0/4 (0.0%)	4/12 (33.3%)	1/2 (50.0%)	3.398	0.393
6mo.	6/12 (50.0%)	1/1 (100.0%)	4/8 (50.0%)	1/2 (50.0%)	1.223	1
9mo.	6/10 (60.0%)	1/1 (100.0%)	5/6 (83.3%)	1/2 (50.0%)	1.993	0.751
12mo.	7/10 (70.0%)	1/1 (100.0%)	5/6 (83.3%)	1/2 (50.0%)	1.742	0.845

Epi, Epigenetics; TRF, Transcriptional Regulatory Factor; STP, Signal Transduction Pathway; SPC, Spliceosome.

### 3.5 Survival and clonal evolution

The median follow-up time was 23 months (3 months to 37.25 months). Nine patients were lost to follow-up, 29 patients were alive, and 8 patients died. Among the 29 surviving patients, 15 patients received IST treatment, 7 patients achieved a PR, 7 patients achieved a CR, and 1 patient had NR and was dependent on blood component transfusion. Among the 8 patients who died, 6 underwent IST. In addition, 3 died from infectious diseases at 1 month (VSAA with WTI variant), 3 months (VSAA with FLT3 variant), and 9 months (VSAA with *ASXL1* and *IDH1* variant) after diagnosis. One SAA patient with an *ETV6* variant died of cerebral hemorrhage 3 months after diagnosis, and one VSAA patient with a *FLT3* variant died of airway bleeding 3 months after diagnosis. In addition, the cause and time of death of one SAA patient with a *BCORL1* variant were unknown. At the end of follow-up, no patient had progressed to PNH or MDS/AML. Univariable survival analysis revealed that disease severity and the hematological response at 3, 6, 9, and 12 months of treatment were significant factors influencing survival time distribution (*P* < 0.05). In contrast, sex, age, disease course, gene variant status and prior HSCT were not significantly associated with survival outcomes. This study has a small sample size. Additionally, There is collinearity in efficacy data during the late-stage follow-up for patients who received treatment; coupled with a reduction in long-term efficacy data, these issues led to abnormal hazard ratios (HR) in the Cox proportional hazards model and meaningless P-values. Combining Kaplan–Meier (K–M) survival analysis with the Cox proportional hazards model, it can be concluded that achieving a hematological response at 3 months after treatment is a key factor that significantly influences survival. (Supplementary Table 2, Supplementary Figure 1).

## 4 Discussion

Currently, the remission rate of AA pediatric patients treated with IST is higher than that of adult patients. Pediatric patients can achieve a relatively high long-term survival rate, but they also experience long-term complications. The likelihood of long-term progression of AA patients to PNH/MDS/AML increases over time, but the cause of this progression remains unclear. Next-generation sequencing has shown that 70% of patients have somatic variants, and in some patients, these somatic variants affect myeloid neoplasm-associated genes (7, 8). This study revealed that patients with myeloid neoplasm-associated gene variants accounted for 20.5% of AA patients diagnosed during the same period. The most commonly mutated genes included *TET2*, *ASXL1* and *MPL*, and the variant rate was higher in the SAA group compared with the NSAA group, which was similar to what has been reported in the literature (9).

On the basis of their mechanisms of action, myeloid neoplasm-associated gene that experienced variants in this group can be divided into epigenetic genes, transcription regulator genes, signal transduction pathway genes, and spliceosome-related genes. The epigenetic genome is closely related to the proliferation or targeted differentiation of hematopoietic cells (10). The epigenetic regulators *ASXL1*, *TET2*, *IDH1*, and *IDH2* regulate chromatin structure (11). The most common somatic variants in AA patients are those of *BCOR* and its ligands *BCORL1*, *DNMT3A*, *TET2*, *ASXL1*, and *PIGA* (12). The risk of clonal transformation to MDS/AML in patients with *ASXL1*, *TP53*, *RUNX1*, and *DNMT3* variants is 40% higher than that in patients with *PIGA*, *BCOR*, and *BCORL1* variants (13). In this study, 43.4% of patients had epigenetic gene variants, most of which were *TET2* and *ASXL1* variants. Among them, 2 patients had 2 epigenetic genes (*ASXL1* and *IDH1*; *BCOR* and *TET2*). The *ASXL1* gene is associated with a poor prognosis in malignant hematological tumors. *TET2*, an epigenetic modifier gene, directly promotes osteogenesis and adipogenesis of bone marrow mesenchymal stem cells and promotes hematopoietic stem cell differentiation into the myeloid lineage (14). Among the transcription regulators, *RUNX1* regulates the transcriptional programs of hematopoiesis. The tumor protein P53 (*TP53*) is a transcription factor that is frequently mutated in cancers and is critical for appropriate cell responses to stress and DNA damage (11). *ETV6* regulates and promotes the differentiation of hematopoietic stem cells through bone marrow mesenchymal stem cells. *TERT* variant can lead to changes in telomerase activity. Leukocyte telomere length is an important predictor of the development of malignant clones and is associated with the recurrence and mortality of aplastic anemia. The shorter the telomeres are, the higher the likelihood of clonal evolution (15). The signal transduction genes *JAK2*, *CALR* and *MPL* are involved in JAK-STAT signaling pathway activation. Activated JAK-STAT signaling is at the core of the pathogenesis of BCR-ABL-negative myeloproliferative neoplasms (MPNs) (11). CBL mutations are rare in AA. In this study, one patient harbored a CBL variant, with the disease phenotype presenting as juvenile myelomonocytic leukemia (JMML). Protein function prediction indicated the variant was deleterious, and pathogenicity analysis suggested it was pathogenic. Both hematological tests and bone marrow examinations of this patient supported the diagnosis of VSAA. Previous studies have shown that CBL variants are associated with susceptibility to JMML (16); however, it remains unclear whether early-stage JMML caused the bone marrow hematopoietic dysfunction in this case. The patient developed the disease at the age of 13 years and 5 months, with a short disease duration and a disease severity of VSAA. HSCT was performed one month after diagnosis, and the patient's blood counts remained normal as of the last follow-up.

In this study, 34.7% (16/46) of the patients had gene variants in signaling pathways, including 5 with *MPL* mutations, 3 with *JAK2*, 3 with *FLT3*, and 3 with *SH2B3*. Mutation of the spliceosome-related gene *SF3B1* is closely related to ineffective erythrocyte formation and is an early event in the development of hematopoietic stem cell (HSPC) dysfunction (17). *SH2B3* is a negative regulator of the JAK-STAT pathway. Some people believe that *SH2B3* variants are related to clonal hematopoiesis (18). In this study, 2 patients had *SF3B1* variants. The KEGG enrichment analysis of genes clearly showed that 20 genes were significantly enriched in key biological pathways related to tumors. These pathways mainly focus on metabolic reprogramming of tumor cells and dysregulation of transcriptional regulatory networks, indicating that these genes play important driving roles in tumor occurrence, development, and maintenance of malignant phenotypes. Studies have found that the gene mutations and signaling pathways that drive the evolution of hematopoietic clones in AA patients significantly overlap with the tumor related metabolic and transcriptional regulatory pathways identified by the KEGG enrichment analysis mentioned above (19).

In this study, protein function prediction indicated 9 patients had pathogenic or likely pathogenic mutations, among whom 8 presented with SAA/VSAA. Pathogenicity analysis identified 5 patients with pathogenic variants, including 3 patients of SAA and 2 patients of AA. Due to the low incidence, the pathogenicity of most genes remains uncertain currently. Additionally, the pathogenicity of different variant sites within the same gene is unclear, requiring further long-term exploration and analysis. The differential diagnosis between AA and hypoplastic myelodysplastic syndrome (hypo-MDS) remains clinically and morphologically challenging. In this study, we identified somatic gene mutations in a subset of pediatric AA patients. Functional validation of the encoded proteins and in silico pathogenicity analyses suggested potential pathogenicity for some of these mutations. However, due to the extremely low incidence of such mutations in pediatric AA, there is currently no consensus or clinical guideline regarding whether they represent true driver mutations or merely passenger mutations. Therefore, the clinical significance of these mutations warrants further validation in larger, prospective studies.

Genetic sequencing can assist in the differential diagnosis of congenital bone marrow failure disorders. However, the comparison of IST efficacy between pediatric AA patients with myeloid neoplasm-associated gene expression and those without requires verification with additional data.One study of 279 AA patients with a median age of 39 years (14–85 years) revealed that somatic variants were not significantly associated with treatment response or long-term survival (20), and a significant correlation was noted between *TET2* variant and a favorable response to IST treatment (21). However, in this study, compared with patients without *TET2* variants, patients with *TET2* variants exhibited no significant differences in the effectiveness of IST treatment for 1, 3, 6, 9, or 12 months. In addition, no deaths occurred among patients with *TET2* variants. *ASXL1* variants are common in AA and MDS/AML, and *ASXL1* variants in AA are associated with clonal evolution and poor prognosis (22, 23). Patients with *PIGA* and *BCOR/BCORL1* variants have better responses to IST treatment and longer OS and progression-free survival (PFS) (22, 24). In this study, 5 patients had *ASXL1* variants, 2 of whom died. Four patients had *BCOR/BCORL1* variants, and 2 patients achieved a PR and a CR at 3 months and 6 months of treatment, separately.

Adult AA patients with PNH clones respond well to IST (25), and one study revealed that 39% of children with AA had detectable PNH clones (26). SAA children who are newly diagnosed with a positive PNH clone have a poorer response to IST and are more likely to develop AA-PNH syndrome (27). In this study, 2 patients exhibited abnormal clones according to FLAER analysis, 1 patient was lost to follow-up, and 1 patient did not progress to PNH after transplantation. By the end of follow-up, no patient had progressed to AML/MDS. However, the follow-up time was short, and long-term follow-up is still needed. For patients with somatic variants, the course of the disease has a significant effect on the risk of MDS transformation (28). Studies have shown that 15%–20% of AA patients develop secondary MDS/AML after 10 years of follow-up (29). However, the probability of AA children developing MDS/AML is much lower than that of adult patients with AA. In a North American study of 314 AA children, 6 (1.9%) patients progressed to MDS/AML (30). In this study, none of the patients developed MDS/AML after 2 years of follow-up. This finding may be related to the short follow-up time; therefore, long-term follow-up is needed.

In conclusion, the majority of myeloid neoplasm-associated gene variants in AA patients affected *TET2*, *ASXL1* and *MPL*, and epigenetic- and signal transduction pathway-related genes were the most commonly involved. No significant differences in IST efficacy were observed in patients with different gene variants. Survival was correlated to the severity of the disease and whether a hematological response was obtained after treatment. Although clonal evolution did not occur, follow-up monitoring still needs to be continued for these patients. This study has limitations including a single-center design, small sample size, and short follow-up duration. While the present study only presents data on gene variants in 46 patients in the figure, subsequent research can be advanced in multiple aspects to enhance its value, considering the limited sample size of pediatric AA and the low incidence of the disease itself. First, the sample size can be expanded through multicenter collaboration, and more in-depth investigations can be conducted in the field of molecular genetics to provide more sufficient evidence for clarifying the clinical significance of these mutations. Second, long-term follow-up of patients is necessary to track their treatment efficacy and outcomes; during follow-up, relevant genetic analyses and next-generation sequencing (NGS) can be supplemented to compare and evaluate dynamic changes in genes. Furthermore, basic experiments can be designed to verify the issues observed in clinical practice, making the research conclusions more persuasive and clinically translatable.

## Data Availability

The original contributions presented in the study are included in the article/Supplementary Material, further inquiries can be directed to the corresponding author.

## References

[1] Scheinberg P. Progress in medical therapy in aplastic anemia: why it took so long? Int J Hematol. (2024) 119:248–54. 10.1007/s12185-024-03713-3 38403842

[2] Groarke EM Patel BA Shalhoub R Gutierrez-Rodrigues F Desai P Leuva H Predictors of clonal evolution and myeloid neoplasia following immunosuppressive therapy in severe aplastic anemia. Leukemia. (2022) 36:2328–37. 10.1038/s41375-022-01636-8 35896822 PMC9701554

[3] Ishiyama K Dung TC Imi T Hosokawa K Nannya Y Yamazaki H Clinical significance of the increased expression of the WT1 gene in peripheral blood of patients with acquired aplastic anemia. EJHaem. (2022) 3:1116–25. 10.1002/jha2.563 36467821 PMC9713059

[4] Chattopadhyay S Lionel S Selvarajan S Devasia AJ Korula A Kulkarni U Relapse and transformation to myelodysplastic syndrome and acute myeloid leukemia following immunosuppressive therapy for aplastic anemia is more common as compared to allogeneic stem cell transplantation with a negative impact on survival. Ann Hematol. (2024) 103:749–58. 10.1007/s00277-024-05621-2 38242970

[5] Fan Y Yin H Hu SY He GS Lu J Xiao PF Analysis of myeloid tumor-related gene mutations in 293 patients with aplastic Anemia. J Clin Pediatr. (2020) 38:647–50. 10.3969/j.issn.1000-3606.2020.09.002

[6] Practice for the Diagnosis and Treatment of Children with Aplastic Anemia. Online publication date: 12/13/2019, Office of National Health Commission of the People’s Republic of China (2019).

[7] Gurnari C Pagliuca S Maciejewski JP. Clonal evolution in aplastic anemia: failed tumor surveillance or maladaptive recovery? Leuk Lymphoma. (2023) 64:1389–99. 10.1080/10428194.2023.2215614 37356012 PMC11104022

[8] Gurnari C Pagliuca S Prata PH Galimard JE Catto LFB Larcher L Clinical and molecular determinants of clonal evolution in aplastic anemia and paroxysmal nocturnal hemoglobinuria. J Clin Oncol. (2023) 41:132–42. 10.1200/JCO.22.00710 36054881 PMC10476808

[9] Zhang T Chen Y Li RX Chen X Long QQ Qiao C Targeted sequencing of genetic mutations in patients with aplastic Anemia. J Clin Hematol. (2021) 34:168–71. 10.13201/j.issn.1004-2806.2021.03.005

[10] Chen ZX. Epigenetic regulation and epigenomic landscape in normal physiological hematopoiesis. J Exp Hematol. (2019) 27:1711–6. 10.19746/j.cnki.issn1009-2137.2019.06.001 31839027

[11] Marneth AE Mullally A. The molecular genetics of myeloproliferative neoplasms. Cold Spring Harb Perspect Med. (2020) 10:a034876. 10.1101/cshperspect.a034876 31548225 PMC6996444

[12] Wirk B. Acquired aplastic Anemia therapies: immunosuppressive therapy versus alternative donor hematopoietic cell transplantation. J Hematol. (2024) 13:61–70. 10.14740/jh1264 38993743 PMC11236356

[13] Piekarska A Pawelec K Szmigielska-Kapłon A Ussowicz M. The state of the art in the treatment of severe aplastic anemia: immunotherapy and hematopoietic cell transplantation in children and adults. Front Immunol. (2024) 15:1378432. 10.3389/fimmu.2024.1378432.eCollection2024 38646536 PMC11026616

[14] Cakouros D Hemming S Gronthos K Liu R Zannettino A Shi S Specific functions of TET1 and TET2 in regulating mesenchymal cell lineage determination. Epigenet Chromatin. (2019) 12:3. 10.1186/s13072-018-0247-4 PMC6317244 30606231

[15] Yuan X Larsson C Xu D. Mechanisms underlying the activation of TERT transcription and telomerase activity in human cancer: old actors and new players. Oncogene. (2019) 38:6172–83. 10.1038/s41388-019-0872-9 31285550 PMC6756069

[16] Maese LD Wlodarski MW Kim SY Bertuch AA Bougeard G Chang VY Update on recommendations for surveillance for children with predisposition to hematopoietic malignancy. Clin Cancer Res. (2024) 30(19):4286–95. 10.1158/1078-0432.CCR-24-0685 39078402 PMC11444884

[17] Ren J Wang HQ Shao ZW. Research progress on SF3B1 mutations in myelodysplastic syndromes. Int J Blood Transfus Hematol. (2023) 46:185–91. 10.3760/cma.j.cn511693-20210222-00036

[18] Ma Q Hu RH Zhao H Lan XX Guo YX Chang XL Genetic variation of SH2B3 in patients with myeloid neoplasms. J Exp Hematol. (2024) 32:1186–90. 10.19746/j.cnki.issn.1009-2137.2024.04.032 39192417

[19] Marsh JC Mufti GJ. Clinical significance of acquired somatic mutations in aplastic anaemia. Int J Hematol. (2016) 104:159–67. 10.1007/s12185-016-1972-8 27084249

[20] Liu L Zhang D Fu Q Wang J Yu J Chen D Clinical implications of myeloid malignancy- related somatic mutations in aplastic anemia. Clin Exp Med. (2023) 23:4473–82. 10.1007/s10238-023-01067-4 37087521 PMC10725342

[21] Li N Liu L Liu Y Luo S Song Y Fang B. miR-144-3p suppresses osteogenic differentiation of BMSCs from patients with aplastic anemia through repression of TET2. Mol Ther Nucleic Acids. (2020) 19:619–26. 10.1016/j.omtn.2019.12.017 31945725 PMC6965517

[22] Zhang ML Chen WS Han B. Somatic mutations of acquired aplastic Anemia. Acta Acad Med Sin. (2022) 44:491–6. 10.3881/j.issn.1000-503x.13381 35791949

[23] Asada S Fujino T Goyama S Kitamura T. The role of ASXL1 in hematopoiesis and myeloid malignancies. Cell Mol Life Sci. (2019) 76:2511–23. 10.1007/s00018-019-03084-7 30927018 PMC11105736

[24] Yoshizato T Dumitriu B Hosokawa K Makishima H Yoshida K Townsley D Somatic mutations and clonal hematopoiesis in aplastic anemia. N Engl J Med. (2015) 373:1673–6. 10.1056/NEJMc1509703 PMC7478337 26132940

[25] Fattizzo B Ireland R Dunlop A Yallop D Kassam S Large J Clinical and prognostic significance of small paroxysmal nocturnal hemoglobinuria clones in myelodysplastic syndrome and aplastic anemia. Leukemia. (2021) 35:3223–31. 10.1038/s41375-021-01190-9 33664463 PMC8550969

[26] Shimano KA Rothman JA Allen SW Castillo P de Jong JLO Dror Y Treatment of newly diagnosed severe aplastic anemia in children: evidence-based recommendations. Pediatr Blood Cancer. (2024) 71:e31070. 10.1002/pbc.31070 38757488

[27] Li J Zong SY Yin ZX Gao YY Liu LP Wan Y Significance of paroxysmal nocturnal hemoglobinuria clone in immunosuppressive therapy for children with severe aplastic Anemia. Chin J Contemp Pediatr. (2022) 24:303–8. 10.7499/j.issn.1008-8830.2110109 PMC8974654 35351262

[28] Kulasekararaj AG Jiang J Smith AE Mohamedali AM Mian S Gandhi S Somatic mutations identify a subgroup of aplastic anemia patients who progress to myelodysplastic syndrome. Blood. (2014) 124:2698–704. 10.1182/blood-2014-05-574889 25139356 PMC4383793

[29] Sun L Babushok DV. Secondary myelodysplastic syndrome and leukemia in acquired aplastic anemia and paroxysmal nocturnal hemoglobinuria. Blood. (2020) 136:36–49. 10.1182/blood.2019000940 32430502 PMC7332901

[30] Rogers ZR Nakano TA Olson TS Bertuch AA Wang W Gillio A Immunosuppressive therapy for pediatric aplastic anemia: a north American pediatric aplastic Anemia consortium study. Haematologica. (2019) 104:1974–83. 10.3324/haematol.2018.206540 30948484 PMC6886407

