# Supplementary material for: Molecular classification and outcomes in pediatric aplastic anemia with myeloid neoplasm-associated gene variants
Source: Front Pediatr. 2025 Dec 16;13:1700402. doi: 10.3389/fped.2025.1700402 (PMC12750608; doi:10.3389/fped.2025.1700402)
Supplement: Supplementary file 1 [file Supplementaryfile1.docx]

Supplementary Material

**Supplementary Table 1** **Hematologic response among different types of aplastic anemia**

| Variable | NSAA | SAA | VSAA | χ² | *P* |
| --- | --- | --- | --- | --- | --- |
| 1mo. | 2/11(18.2%) | 1/14(7.1%) | 0/17(0.0%) | 3.022 | 0.172 |
| 3mo. | 5/11(45.5%) | 2/11(18.2%) | 5/14(38.9%) | 1.887 | 0.376 |
| 6mo. | 4/9(44.4%) | 4/10(40.0%) | 5/8(62.5%) | 1.027 | 0.708 |
| 9mo. | 5/9(55.6%) | 4/6(66.7%) | 5/6(83.3%) | 1.252 | 0.837 |
| 12mo. | 6/9(66.7%) | 4/6(66.7%) | 5/6(83.3%) | 0.708 | 0.851 |

NSAA,nonsevere aplastic anemia; SAA, severe aplastic anemia; VSAA, very severe aplastic anemia

**Supplementary Table 2 Univariate analysis and Multivariate analysis of survival time in AA patients**

| Variables | **Univariate** | | | **Multivariate** | | |
| --- | --- | --- | --- | --- | --- | --- |
|  | *95%CI* | χ² | *P* | *HR* | *95%CI* | *P* |
| Gender |  | 0.026 | 0.873 |  |  |  |
| Male | 16.80-39.13 |  |  | - | - |  |
| Female | 30.31-41.69 |  |  | 0.798 | 0.05-12.89 | 0.874 |
| Age |  | 1.740 | 0.419 |  |  |  |
| 0-3y | 13.19-70.81 |  |  |  |  |  |
| 3-5y | 22.50-43.50 |  |  |  |  |  |
| ≥5y | 8.66-103.34 |  |  |  |  |  |
| Course of Disease |  | 2.136 | 0.344 |  |  |  |
| ＜1mo. | 16.64-39.36 |  |  |  |  |  |
| 1mo.-3mo. | 17.07-66.94 |  |  |  |  |  |
| ≥3mo. | 15.16-30.84 |  |  |  |  |  |
| Number of Genes |  | 2.855 | 0.091 |  |  |  |
| 1 | 26.05-45.95 |  |  |  |  |  |
| ＞1 | 10.53-37.47 |  |  |  |  |  |
| Gene mutation |  | 5.325 | 0.256 |  |  |  |
| Epi | 19.14-46.86 |  |  |  |  |  |
| TRF | 11.38-58.62 |  |  |  |  |  |
| STP | 20.66-57.34 |  |  |  |  |  |
| SPC | 36.00-36.00 |  |  |  |  |  |
| Two types | 09.60-38.40 |  |  |  |  |  |
| Degree of BMF |  | 5.532 | 0.063 |  |  |  |
| NSAA | 17.52-94.48 |  |  |  |  |  |
| SAA | 30.65-47.35 |  |  |  |  |  |
| VSAA | 18.34-29.66 |  |  |  |  |  |
| HR(1mo.) |  | 0.021 | 0.886 |  |  |  |
| N | 23.37-42.63 |  |  | - | - |  |
| Y | 36.00-36.00 |  |  | 113.06 | 0.98-13485 | 0.053 |
| HR(3mo.) |  | 9.190 | 0.002 |  |  |  |
| N | 28.70-55.30 |  |  |  |  |  |
| Y | 14.37-31.63 |  |  |  |  |  |
| HR(6mo.) |  | 10.918 | 0.001 |  |  |  |
| N | 34.64-77.37 |  |  |  |  |  |
| Y | 15.78-32.22 |  |  |  |  |  |
| HR (9mo.) |  | 11.416 | 0.001 |  |  |  |
| N | 68.00-68.00 |  |  |  |  |  |
| Y | 14.83-33.17 |  |  |  |  |  |
| HR (12mo.) |  | 11.176 | 0.001 |  |  |  |
| N | 68.00-68.00 |  |  |  |  |  |
| Y | 19.01-36.99 |  |  |  |  |  |
| HSCT |  | 0.467 | 0.495 |  |  |  |
| N | 21.26-44.74 |  |  |  |  |  |
| Y | 12.83-53.17 |  |  |  |  |  |

Epi, Epigenetics; TRF, Transcriptional Regulatory Factor; STP, Signal Transduction Pathway; SPC, Spliceosome; mo., Month;HR, hematological response;HSCT, hematopoietic stem cell transplantation


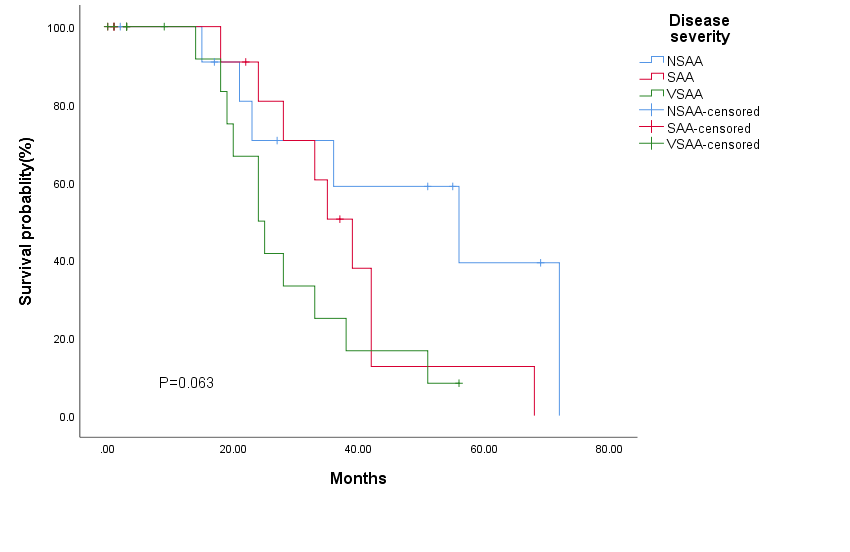

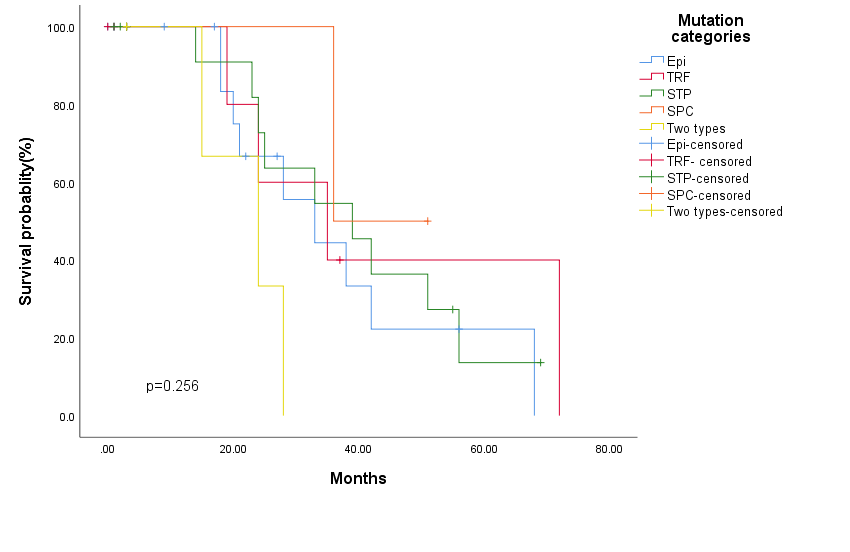


**b**

**a**


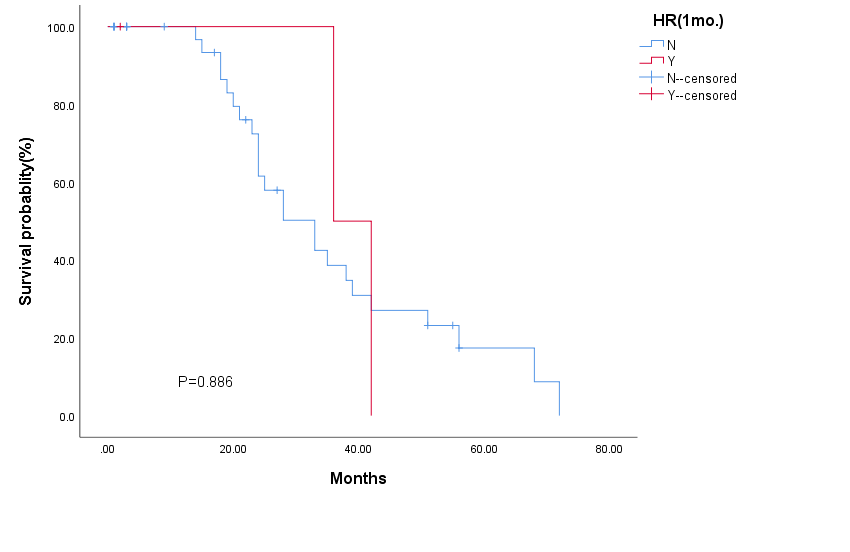

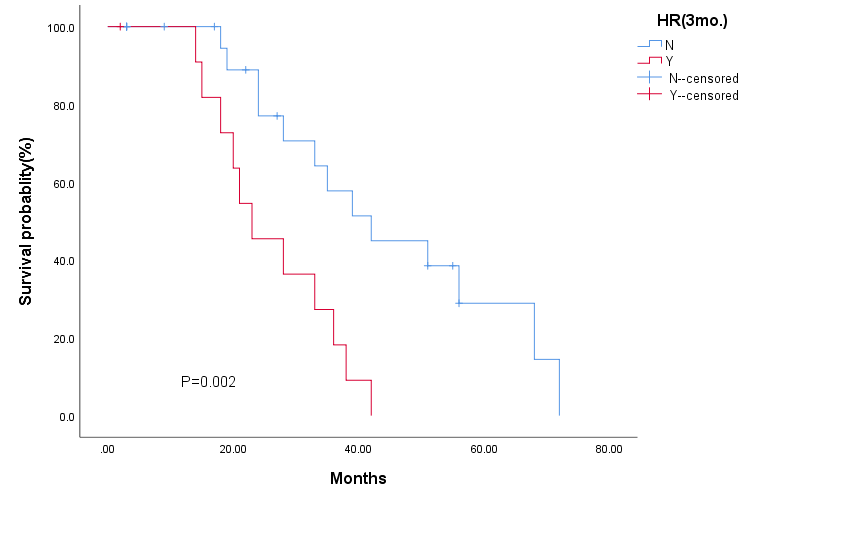


**C2**

**C1**


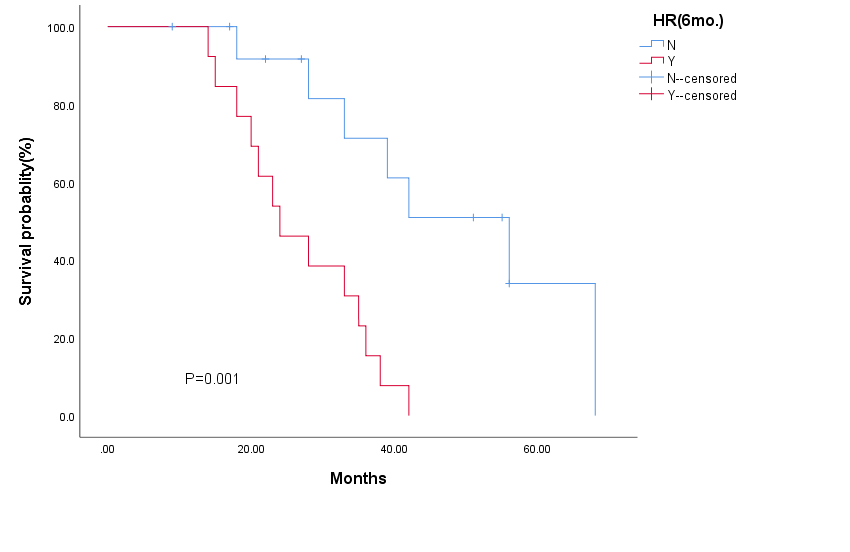

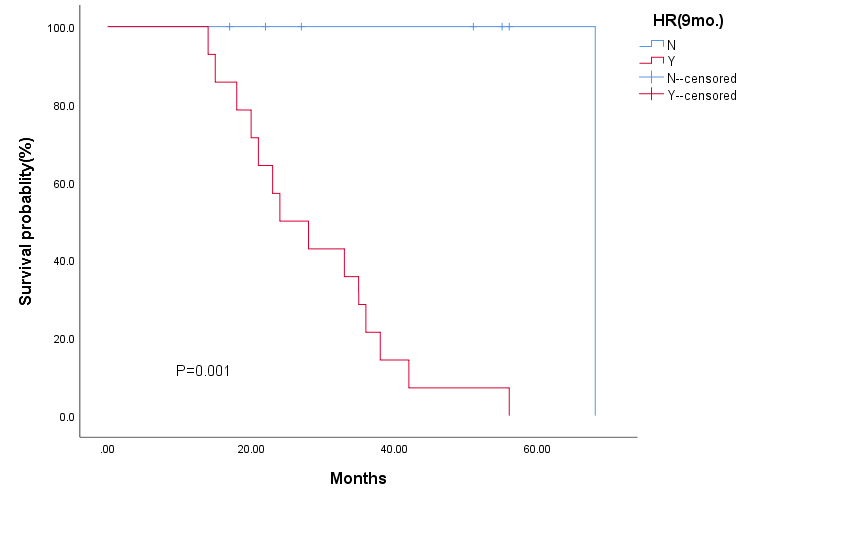


**C4**

**C3**


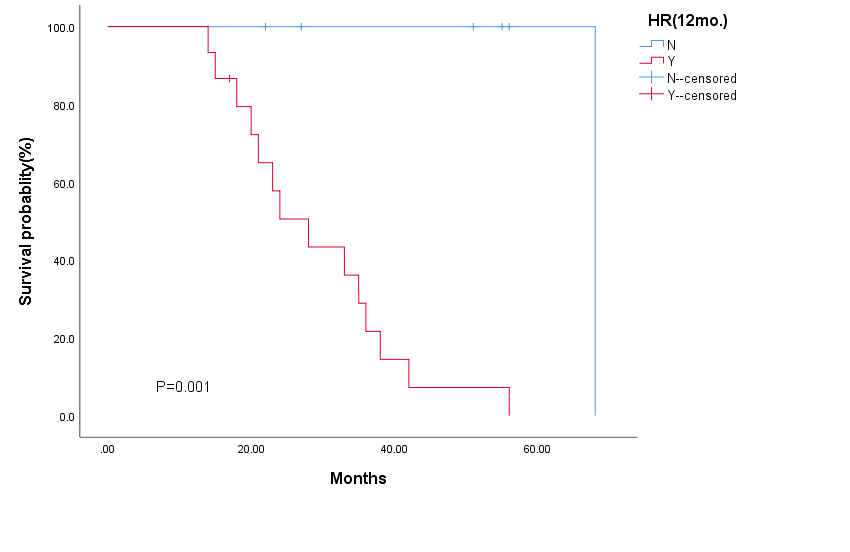


**C5**

**Supplementary Figure 1 Kaplan-Meier survival curve in AA patients, (a), Disease Severity;** **(b), Mutation Categories; (c)** **Hematological Reactions (HR).**
